# Alcohol Septal Ablation: An Option on the Rise in Hypertrophic Obstructive Cardiomyopathy

**DOI:** 10.3390/jcm10112276

**Published:** 2021-05-24

**Authors:** Victor Arévalos, Juan José Rodríguez-Arias, Salvatore Brugaletta, Antonio Micari, Francesco Costa, Xavier Freixa, Mónica Masotti, Manel Sabaté, Ander Regueiro

**Affiliations:** 1 Department of Cardiology, Clinic Cardiovascular Institute, Hospital Clinic de Barcelona, 08036 Barcelona, Spain; varevalos88@gmail.com (V.A.); juanjose.rodriguez.a@gmail.com (J.J.R.-A.); sabrugaletta@gmail.com (S.B.); freixa@clinic.cat (X.F.); masotti@clinic.cat (M.M.); masabate@clinic.cat (M.S.); 2 Institut d’Investigacions Biomèdiques August Pi i Sunyer (IDIBAPS), University of Barcelona, 08036 Barcelona, Spain; 3 Interventional Cardiology Unit, Policlinic G. Martino, University of Messina, 98124 Messina, Italy; antonio.micari@unime.it (A.M.); dottfrancescocosta@gmail.com (F.C.)

**Keywords:** hypertrophic obstructive cardiomyopathy, alcohol septal ablation, septal myectomy

## Abstract

Hypertrophic cardiomyopathy (HCM) can cause symptoms due to the obstruction of the left ventricle outflow tract (LVOT). Although pharmacological therapy is the first step for treating this condition, many patients do not fully respond to the treatment, and an invasive approach is required to manage symptoms. Septal reduction therapies include septal myectomy (SM) and alcohol septal ablation (ASA). ASA consists of a selective infusion of high-grade alcohol into a septal branch supplying the basal interventricular septum to create an iatrogenic infarction with the aim of reducing LVOT obstruction. Currently, SM and ASA have the same level of indication; however, ASA is normally reserved for patients of advanced age, with comorbidities or when the surgical approach is not feasible. Recent data suggests that there are no differences in short- and long-term all-cause mortality, cardiovascular mortality and sudden cardiac death between ASA and SM. Despite the greater experience and refinement of the technique gained in recent years, the most common complication continues to be complete atrio-ventricular block, requiring a permanent pacemaker. Septal reduction therapies should be performed in experienced centres with comprehensive programs.

## 1. Introduction

Hypertrophic cardiomyopathy (HCM) is defined by an unexplained left ventricular hypertrophy, not solely secondary to abnormal loading conditions. It is a common disease with a reported prevalence of 1:500 individuals. The etiology is usually genetic, with up to 60% of cases being associated with an autosomal dominant trait caused by mutations in cardiac sarcomere protein genes [1,2].

Among patients with HCM, left ventricle outflow tract obstruction (LVOTO) is present in one third of patients at rest and in another third during exertion, strain due to the Valsalva maneuver or after pharmacological stress, giving rise to the term hypertrophic obstructive cardiomyopathy (HOCM). LVOTO is conventionally defined as an instantaneous LV Doppler peak outflow tract gradient of ≥30 mm Hg, but the threshold for invasive treatment is ≥50 mm Hg. Despite many patients being asymptomatic, a variable proportion of them may develop symptoms related to LVOTO or arrhythmic events [1,2,3].

The obstruction mechanism is usually the combination of basal septal hypertrophy and systolic anterior motion (SAM) of the mitral valve. LVOTO is one of the main factors related to morbidity and mortality [4]. When the patient presents symptoms, such as dyspnea, angina, or syncope, which can be attributed to this anatomical feature, the initial approach is pharmacological treatment.

Medical therapy aims to reduce the obstruction gradient and arrhythmia. Reduction of the LVOTO is mainly achieved by means of cardio-selective beta blockers due to their negative inotropic effect, which, in turn, also have antiarrhythmic properties [5]. Other possibilities are verapamil or disopyramide in combination with beta blockers; however, this combination is usually not tolerated due to the anticholinergic side effects [6]

In patients with HOCM who remain severely symptomatic despite guideline-directed medical treatment, invasive treatment or septal reduction therapy (SRT) must be performed.

## 2. Septal Reduction Therapy

SRT is mainly indicated by both the American and European guidelines in cases of persistence of symptoms, despite optimized drug therapy (NYHA class III–IV), and a LVOTO gradient ≥ 50 mmHg. There are two main options: surgical and percutaneous treatment [1,2].

**Figure 1:**
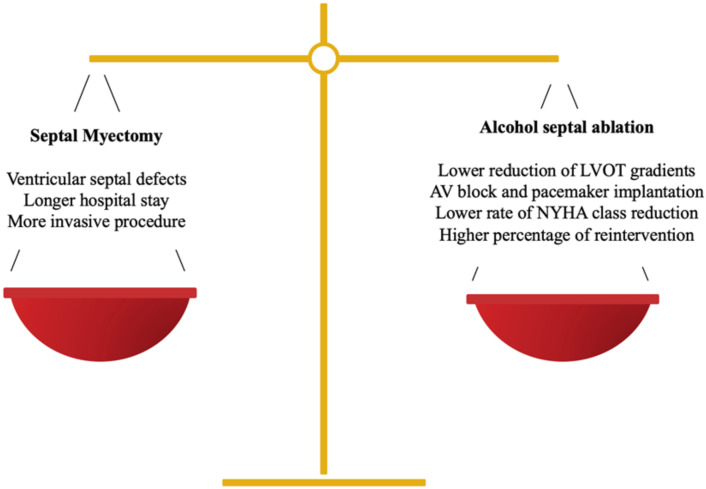
Balance between SM and ASA in the decision-making process.

Treatment must be individualized, taking into account the anatomic structure, the functional conditions and the patient’s wishes, via a multidisciplinary approach [7]. When making decisions, not only the advantages, but also the disadvantages of each technique must be considered (Figure 1). At present, both invasive options, i.e., septal myectomy (SM) and alcohol septal ablation (ASA), have a recommendation class I in the European and American guidelines [1]. It is worth mentioning that personal preferences currently play a crucial role in the decision-making process. The results of both techniques are largely dependent on the center’s experience. A cardiomyopathy team, comprising a cardiologist expert in HOCM, an interventional cardiologist and a surgeon specialized in ASA and SM respectively, is recommended for evaluations of each patient in the decision-making process [2].

The best results are achieved in centers with a high volume of procedures, both in SM and ASA. Mortality in SM can be as high as 4–16% compared with that of high volume centers (1%), and in the case of ASA, a lower rate of complications and better survival rates are also observed [8]. American guidelines recommend performing these procedures in reference centers with a very low mortality rate and infrequent major complications [2]. If this is not possible, a reasonable number of at least 10 procedures per year for both SM and ASA is recommended [9].

### 2.1. Surgical Approach

Even though HCM was initially described in 1958 by D. Teare, the first surgical procedure for its treatment was performed via the aortic root in 1961 [10]. Surgical treatment is based on SM in order to reduce the LVOTO gradient. This procedure is accompanied by surgical treatment of the mitral valve in 10 to 11% of patients, but realignment of papillary muscles and valve plication may also be necessary [11,12].

Morrow’s modified SM is the preferred technique, mostly in young patients or in the presence of other pathologies requiring concomitant treatment, such as mitral valve anomalies or coronary disease.

The worst complication in the surgical treatment of HOCM is ventricular septal defects, and the main risk factors are multiple concurrent surgical treatments (e.g., myectomy and coronary bypass grafting) and septum thickness < 20 mm [13]. In addition, aortic valve regurgitation due to lack of septal support or septal coronary arteries dissection could also occur [14]. New regurgitation jets after surgical treatment should be carefully evaluated to rule-out these complications.

The surgical approach affords great success in restoring quality of life, with reduction of LVOTO in 90% of the cases and low mortality rates [15]. SM mortality rates range from 1 to 2% [16,17,18]. Long-term survival of patients treated surgically is comparable to that of the general population and, in addition, a secondary benefit of reduced risk of sudden cardiac death has been observed [18,19].

### 2.2. Percutaneous Approach

Furthermore, ASA has the same level of indication of SM according to European and American guidelines. However, the ACC/AHA guidelines establish surgical treatment as the first option, reserving ASA for patients for whom surgery is contraindicated or the risk is considered unacceptable due to severe comorbidities or advanced age. (Table 1) [1,2]

ASA consists of a selective infusion of high-grade alcohol into a septal branch supplying the basal interventricular septum to create an iatrogenic infarction with the aim of reducing the LVOT gradient. This procedure is less invasive than surgical myectomy, and requires a shorter hospital stay [20]. However, a higher rate of arrhythmic episodes could be related to this technique after the procedure, and in the long term, due to the creation of scar tissue [21]. Also, the results are visible three to six months after the procedure, as this is the time it takes the myocardium to reduce after cell necrosis.

The success of this technique, similar to the surgical approach and other percutaneous procedures, is operator dependent and, in high volume centers, mortality is as low as 1% [22]. There is a steep learning curve for the optimal performance of ASA. Despite there being less difference in the outcome of ASA between low- and high-volume centers, the optimal results of ASA are from highly experienced operators. [23,24]

As main findings, Bytyçi et al. observed a higher rate of pacemaker implantation, higher rates of reintervention and less improvement of clinical symptoms in ASA compared with SM, but in exchange, ASA patients presented lower periprocedural complications [25].

## 3. ASA Technique and Considerations

### 3.1. Anatomical Considerations

A careful structural evaluation should be carried out in patients considered candidates for SRT. It is mandatory to perform a systematic assessment of the LVOT, the septum and mitral valve to exclude other LVOT obstruction mechanisms, and mitral valve abnormalities requiring surgical treatment. [1,2] The contraindications for ASA are anatomical or structural conditions that limit the safety and efficacy of the technique, which cannot be resolved appropriately via a percutaneous approach or which make surgical treatment preferable (Table 2).

Septum morphology must be known in order to predict the feasibility of ASA. The technique may be less effective in cases with extensive septal scarring via cardiac magnetic resonance (CMR), and in patients with very severe hypertrophy (≥30 mm), but there is a lack of data confirming this. Whereas, patients with mild hypertrophy (≤16 mm) are at high risk of septal defects with any SRT [1,26].

The coronary anatomy and concomitant atherosclerotic coronary disease should be assessed before the ASA procedure (even without angina or ischemic symptoms) by invasive coronary angiography [27]. However, in some centers, coronary angiography is performed during the same ASA procedure. Identifying the most suitable septal branch for ablation is essential for the viability of the procedure. In most cases, the suitable artery is the first septal, which commonly arises from the left anterior descending (LAD). However, the first septal sometimes arises from the diagonal, ramus intermedius, left main (LM) or even from the right coronary artery (RCA) [28].

### 3.2. Role of Echocardiography in ASA

One of the most important advances towards a greater safety and efficacy for this technique was the addition of periprocedural echocardiographic monitoring [29]. Transthoracic echocardiography (TTE) during the intervention avoids the use of double arterial access or transseptal route for gradient measurement, and increases the likelihood of success by selecting the septal branch (or branches) most suitable for treatment.

TTE is performed with the use of intra-coronary injection of echocardiographic contrast, helping to visualize the myocardial area perfused from the septal vessel. Different echocardiographic contrasts can be used. Also, Gelafundin^®^, a volume expander has been shown to be good for generating myocardial enhancement. The echo-bright contrast should enhance the proximal hypertrophied septum at the point of maximal systolic anterior motion of the anterior mitral valve leaflet with associated septal contact. Also, this tool has played an essential role in ASA, minimizing periprocedural complications [1,30]. Due to the anatomical variations of the territory perfused by the first septal, the area supplied by this vessel may include a variable quantity of myocardial tissue, which could involve apart the hypertrophied basal septum, also the right ventricular septum, free wall and even the apex of the LV. Patients with a large area of myocardium at risk, or who have a significant involvement of the right ventricle, have a higher complication rate, including complete AV block [31]. Knowledge regarding the specific myocardial area perfused by the septal allows us to avoid ablation, which can affect unwanted or potentially dangerous territories [32,33]. The use of TTE was improved in recent years via the three-dimensional assessment of the myocardial distribution of the contrast, this technique can be used in some challenging cases to achieve a better visualization of the enhanced septum [34].

### 3.3. Step-by-Step and Technical Considerations

**Figure 2:**
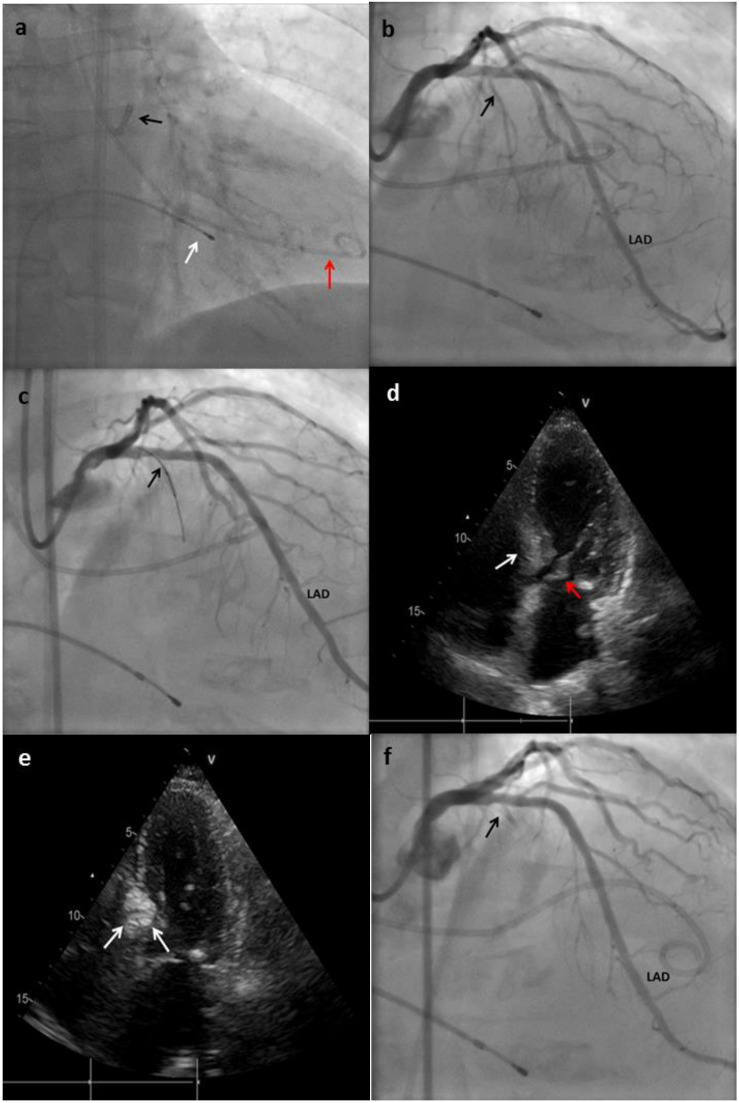
Main steps of the ASA technique performed in a patient at our center. (**a**) An extra back-up (EBU) catheter was placed on the ostium of the left main (black arrow); a pig-tail catheter was placed in the left ventricle (LV) for gradient monitoring (red arrow); temporary pacemaker (PM) electro-catheter was placed on the right ventricle (RV) (white arrow). (**b**) Coronary angiogram showing absence of atheroscletic lesions and the origin of the first septal branch (black arrow). LAD: left anterior descending. (**c**) The first septal branch is wired and an over-the-wire (OTW) balloon is advanced to this artery (black arrow). (**d**) Transthoracic echocardiography (TTE) showing hypertrophy of the basal septum (white arrow), which makes contact during systole with the anterior leaflet of mitral valve (red arrow). (**e**) TTE performed after Gelafundin^®^ (B. Braun, Melsungen, Germany) injection in the septal branch; enhancement of the basal septum can be seen (white arrows), this indicates that the first septal is suitable for ablation. (**f**) Coronary angiogram showing final result with occlusion of the first septal branch after alcohol injection.

The technique for performing ASA differs between centers in the minor details, but the main steps have remained similar since the first description of the procedure (Figure 2). The procedure is carried out with conscious sedation to preserve accurate hemodynamics, vital for the correct estimation of the changes in the gradients during ablation [28].

(1) The first step is to obtain an arterial access with a 6 or 7 F sheath, radial or femoral. At the beginning, femoral was the most common access, with a change in the approach in recent years. Radial access was proposed to achieve less patient discomfort, early ambulation, and less vascular complications [35]. Depending on the preferences of the center, two accesses could be performed, one for the catheter used for ablation, and the other for an invasive monitoring of the gradient during the procedure.
(2) The common femoral vein (or internal jugular vein) is punctured and a temporary pacemaker (PM) is placed on the right ventricle. This step is important to ensure a fast response in case of complete atrio-ventricular (AV) block [36].
(3) After vascular accesses are obtained, 100 units per 1 kg of weight of unfractionated heparin should be administrated.
(4) LM ostium should be engaged with standard guide catheters 6–7 F (Judkins left, Amplatz left or Extra Back-Up). If invasive monitoring is performed during the procedure, a pigtail catheter is placed into the left ventricle (LV) via a second arterial access.
(5) The gradient present through the LVOT must be measured using TTE and/or the catheters placed in the aorta and LV.
(6) Coronary angiography is performed to identify a suitable septal branch. The trajectory of the septal arteries can be visualized through right anterior oblique or postero-anterior cranial projections, while the left anterior oblique view allows differentiating whether the septal branches run on the right or left side of the septum.
(7) A 0.014-inch coronary guidewire is advanced to the first septal artery and, through this, an over-the-wire (OTW) balloon is placed in this vessel. In some cases, the takeoff of the septal vessel is extremely angulated, making it difficult to advance the balloon; when this situation occurs, an extra support wire can be used. The size of the balloon should generally be short (1.5–2.5 mm in diameter, 6–10 mm in length, and with a balloon-artery ratio of 1.3:1 approximately or usually 0.25 mm greater than the vessel diameter). Short balloons are recommended to avoid hyperselectivity in the presence of a septal branch with early bifurcation [26,37]. After placing the balloon, the guidewire is removed.
(8) The balloon is inflated at low pressure (5 or 10 atm), and then slow injection of 1–2 mL of angiographic contrast should be performed to test the correct occlusion of the septal, absence of contrast reflux into the LAD, and to rule out the presence of collateral flow from the septal branch toward another branch of the left or right coronary system [26,29]. During balloon inflation, continuous monitoring of the gradient could show a significant reduction, indicating a favorable target vessel and generally predicts a good response to ASA [27].
(9) Echocardiographic contrast is injected through the OTW balloon lumen. The possible area affected by the ablation is evaluated. If the contrast medium enhances an inadequate territory for ASA, the balloon must be deflated and repositioned in another branch. In some cases, it is not possible to identify a suitable vessel that perfuses the base of the hypertrophied septum at the point where maximal systolic anterior motion occurs, in which case the procedure should be discontinued [27]. Inability to identify a satisfactory septal branch occurs in approximately 10% of the candidates.
(10) Having selected the septal target vessel via the prior angiographic and echocardiographic assessment, ˃94° ethanol is injected through the OTW balloon lumen into the branch. The amount of ethanol should be of 1 to 3 mL [1]. Some authors describe an efficient and safe way to measure the quantity of ethanol as 0.1 mL per 1 mm of septal thickness. Higher doses were associated with higher rates of complications and postprocedural mortality [38]. Potential explanations could lie in the more extended infarct scar due to the higher alcohol dose. Ethanol must be injected slowly, usually 1 min per ml. A slow injection, rather than a bolus administration was demonstrated to be safer [27,39].
(11) Analgesics (i.e., morphine) can be administrated to avoid the pain caused by the iatrogenic myocardial infarction.
(12) After instillation, balloon occlusion should be maintained for at least 3 to 5 min. The catheter is flushed with saline before the balloon is deflated and removed from the septal branch to prevent spillage of alcohol into the LAD circulation [27].
(13) The balloon is removed, and a coronary angiogram is performed to ensure septal branch no-reflow and to rule out any unexpected complication.
(14) Continual monitoring is used to measure the effects of ASA in gradient values. A gradient reduction ≥ 50% from baseline is considered successful (by echocardiography or invasive hemodynamics). If the ablation fails to achieve this improvement, a second septal or subseptal branch should be explored [28].
(15) Once the objective of gradient reduction has been achieved, or there are no additional septal branches perfusing the area that need ablation, the procedure is terminated. The arterial accesses are removed and subjected to hemostasis, and the temporary PM is secured.

### 3.4. Postprocedural Care

After the procedure is completed, the patient is transferred to the coronary unit for monitoring. The length of stay in this unit is usually three days, one to two days if the patient already had a permanent PM or defibrillator. Subsequently, the patient must remain admitted to the general cardiology ward for 24 to 48 h [28]. Before, Creatine kinase (CK) was measured during this period as a way to predict the efficacy of the ablation. Although a correlation between the ethanol dose and the myocardial necrosis area measured by cardiac biomarkers is recognized, CK levels have not been proven to predict procedural success or LVOT gradient reduction at follow-up [40].

There is no general agreement as to how long the patient should keep the temporary PM. However, it could be removed before transfer from the coronary unit if there is no high-grade or complete AV block.

In the absence of significant bradyarrhythmia, the preprocedural negative inotropic and chronotropic medications could be restarted at a lower dose, especially beta blockers [28].

## 4. Periprocedural Complications

The most common complications of ASA are AV conduction disturbances, with different prevalence in the published series. Right bundle branch block (RBBB) occurs in 37–70% of the patients, while complete AV block requiring permanent pacing occurs in approximately 10–15% of patients after alcohol septal ablation [41]. There are anatomical reasons for this fact. As demonstrated in a previous study with CMR in patients undergoing SRT, ASA can produce a transmural necrosis in the septum in up to 75% of patients. This necrosis more frequently affects the course of the right bundle branch compared with myectomy, and sometimes additional segments of the left bundle branch can be affected, producing a complete AV block [42,43]. This disturbance may be transitory in 10 to 46% of patients with recovery within the first 24 h, while some patients might develop it up to nine days after septal ablation [36,44]. The previous presence of left bundle branch block (LBBB), due to the aforementioned anatomical reasons is the main predictor of complete AV block [36]. Furthermore, the ethanol dose used in the ablation and the experience of the operator could be related to this outcome [45].

Another expected arrhythmic complication, such as ventricular arrhythmias, is less common in the early phases after ASA. There are some reports of ventricular tachycardia and ventricular fibrillation in these patients. However, they remain relatively rare, with many series showing no increased risk of sudden death in the immediate postprocedural period [45,46]. The concerns about these ventricular arrhythmias originate from the known scar tissue consequence of the ablation.

As in other catheter-based coronary interventions, less common complications could be coronary dissection, cardiac tamponade and vascular complications related to access site. The volume of interventions at the center and the operator experience are related to the occurrence of these complications [26]. The radial access was shown to be safer, with less vascular complications, compared with femoral access [35].

Other potential complications of this technique are the infarction of the anterior wall, papillary muscles, or right ventricle due to collateral septal flow to the RCA or LAD artery [47]. Procedure-related death is less than 1% in experienced hands, similar to that of myectomy in high volume surgical centers [48].

## 5. Limitations of the Technique

Although ASA has become a robust therapeutic option over the last 20 years, there are some limitations with this technique that are worth mentioning. The first and most important is the persistent high incidence of complete AV block with the need for permanent PM [41]. Second, there is limited data regarding long-term results. Furthermore, there are no randomized clinical trials comparing the results of ASA vs. SM. Third, ASA is totally dependent on the coronary anatomy, and the impossibility of identifying a satisfactory septal branch occurs in approximately 10% of the candidates [26,42].

## 6. Long-Term Results

A recent meta-analysis has shown no differences between short-term and long-term all-cause mortality, cardiovascular mortality and sudden cardiac death compared to SM. However, compared with SM, septal ablation has been associated with a lower decrease in the LVOT gradient, less improvement in symptoms, higher pacemaker implantation and a higher frequency of reinvention. [25,37,49]

ASA presents a high long-term survival with a series that showed a five-year survival of 98.9% [50]. A study that evaluated the efficacy of ASA in the very long term demonstrated a high survival, persistent improvement in functional class and low peak resting gradients. At 10 year follow-up, survival seems similar to those patients without obstructive cardiomyopathy, with very low periprocedural mortality (0.89%). [51,52]. Reduction of symptoms, both dyspnea and angina, appears during the first year after procedure and stabilize with no further improvement in the long-term [53]. Recently, in the Euro-ASA registry that included 1275 patients treated with ASA and no mitral valve disease, this technique presented a 1% mortality during the first month, 89% of the patients had mild or no symptoms (NYHA class < 2), however a high rate of pacemaker implantation of 12% was observed [54]. Focusing on older patients, who seem to obtain the best benefits from this technique, Jahnlová et al., presented their study on patients over 60-years of age. The mortality rate was 2.6% during the first month and with greater pacemaker implantation than previous studies (11.5%), but 81% of patients presented with mild or no dyspnea symptoms [55]. These results are similar to the ASA registry.

Different studies have looked for markers of prognosis after the ASA procedure. Improvement in functional activity measured by the treadmill test at three months seems to be related to better long-term outcomes regarding symptomatology [56]. Imaging studies, predominantly CMR, can help to establish the scar tissue developed after septal ablation, which is related to LVOT gradient reduction [57]. Finally, ASA responders present a greater decline in brain natriuretic peptide levels at three months that generally stabilize at the one-year follow-up [58].

## 7. Myosin Inhibitors: Perspectives of a Future Option

New potential therapeutic options for the treatment of HCM are currently under study. Among these options is a new class of drugs, myosin inhibitors. The mechanism of action of these drugs is based on blocking the ATP-converting enzyme on the myosin head, which normalizes myosin activation and causes reversible inhibition of actin-myosin cross bridging to reduce contractility [59].

Mavacamten is the myosin inhibitor that has been the most studied to date. In the phase III EXPLORER HCM clinical trial, a total of 251 patients with HCM, LVOT gradient ≥ 50 mmHg and NYHA class II–III symptoms were randomly assigned to receive mavacamten (starting at 5 mg) or placebo for 30 weeks. At the end of the follow-up, it was found that mavacamten improved exercise capacity, LVOT obstruction, NYHA functional class, and health status compared with the placebo group. However, due to its negative inotropic mechanism, approximately 10% of patients experienced a significant transient decrease in ejection fraction to <50%, highlighting the need for longer follow-up to better understand the safety profile of mavacamten [60].

## 8. Conclusions

Septal reduction therapies including SM and percutaneous ASA are established invasive strategies to reduce LVOT obstruction and improve symptoms in selected patients with HCM. ASA has proven to be a safe and effective, less-invasive alternative to SM in selected patients if performed in experienced centers with sufficient volume and trained personnel. Yet, inherent complications, such as complete AV block with the need for permanent PM implantation remain frequent after ASA, and should be the basis for a shared decision-making discussion with the patient at the time of procedure selection.

## Figures and Tables

**Table 1:** Factors in decision-making for the invasive treatment of HOCM.

	Favors SM	Favors ASA
Clinical factors		
Young age	+	
Advanced age		+
High surgical risk/severe comorbidity		+
Frailty		+
Cardiac conditions		
Previous cardiac surgery		+
Previous pacemaker or defibrillator		+
Right bundle branch block		+
Left bundle branch block	+	
Mid-ventricular obstruction	+	
Operator related factors		
Local operator experience in SM	+	
Local operator experience in ASA		+
Patient’s preference	±	±

ASA, alcohol septal ablation; SM, septal myectomy.

**Table 2:** ASA: indications and contraindications.

Indications	Contraindications
Severe symptoms (NYHA III-IV, CCS III-IV angina, presyncope, or recurrent syncope) despite GDMT.	Presence of a supra or subvalvular aortic membrane
LVOT gradient ≥ 50 mm Hg at rest or with provocation despite maximum-tolerated medical treatment	Severe coronary artery disease requiring coronary artery bypass graft surgery
At least one septal artery supplying the target septal area (left ventricular outflow tract obstruction zone)	Severe aortic stenosis requiring surgical valve replacement
Life expectancy > 1 year, absence of comorbidities that would compromise clinical improvement (i.e., severe dementia)	Severe valvular or mitral valve abnormality requiring surgical treatment
	Septal thickness > 30 mm or ≤16 mm

CCS, Canadian Cardiovascular Society; GDMT, guide-directed medical therapy; NYHA, New York Heart Association; LVOT, left ventricle outflow tract.

## Data Availability

Not applicable.
